# Effects of hemoadsorption on plasma catecholamine levels: an in vitro study

**DOI:** 10.1038/s41598-026-49101-1

**Published:** 2026-04-20

**Authors:** Andreas Körtge, Gerd Klinkmann, Christoph Kamper, Reinhold Wasserkort, Steffen Mitzner

**Affiliations:** 1 Department of Extracorporeal Therapy Systems, Fraunhofer IZI, Fraunhofer Institute for Cell Therapy and Immunology IZI, Rostock, Germany; 2 Center for Internal Medicine, Division of Nephrology, Rostock University Medical Center, Rostock, Germany; 3 Department of Anaesthesiology, Intensive Care Medicine and Pain Therapy, Rostock University Medical Center, Rostock, Germany; 4 International Renal Research Institute of Vicenza, Vicenza, Italy

**Keywords:** Hemoadsorption, CytoSorb, Catecholamines, Epinephrine, Norepinephrine, Dobutamine, Sepsis, Septic shock, Drug discovery, Medical research

## Abstract

Hemoadsorption with polystyrene-divinylbenzene (PS-DVB)-based adsorbents is explored as an adjunctive extracorporeal therapy in selected critically ill patients with dysregulated systemic inflammation. However, such therapy must not interfere with standard treatments, including antimicrobial therapy and hemodynamic stabilization with vasoactive drugs. As catecholamines are rarely quantified analytically in clinical practice, there are currently no studies reporting actual plasma levels during hemoadsorption. This in vitro study evaluated whether hemoadsorption with PS-DVB-based adsorbents alters plasma concentrations of epinephrine, norepinephrine, and dobutamine. Miniaturized PS-DVB adsorbers were used in 300-minute experiments; serial plasma samples were quantified, and apparent plasma clearance was derived from concentration-time data. Epinephrine and norepinephrine concentrations remained essentially unchanged over 300 min, with very low apparent plasma clearance (< 5 mL/min), indicating no meaningful removal under the tested conditions. By contrast, dobutamine demonstrated consistent, measurable clearance (approximately 5–15 mL/min), indicating non-negligible adsorption. Under these in vitro conditions, PS-DVB hemoadsorption did not materially remove epinephrine or norepinephrine but produced measurable removal of dobutamine. These findings are compatible with observational reports of reduced overall vasopressor requirements during hemoadsorption, while highlighting the potential for dobutamine underexposure. Future clinical studies should directly measure therapeutic plasma levels – particularly for dobutamine – during PS-DVB hemoadsorption and assess whether dosing adjustments are required to maintain intended inotropic effects without compromising safety.

**Supplementary Information:**

The online version contains supplementary material available at 10.1038/s41598-026-49101-1.

## Introduction

Hemoadsorption using polystyrene-divinylbenzene (PS-DVB) adsorbents is being evaluated as an adjunctive extracorporeal option to modulate the dysregulated host response in septic shock by binding circulating mediators within a middle-molecular-weight window. Contemporary reviews describe the resin’s selective but non-specific, largely hydrophobic adsorption characteristics and operational feasibility, while emphasizing that clinical application should complement and not replace guideline-directed sepsis care^1–3^. Observational studies and expert syntheses report hemodynamic signals during hemoadsorption (e.g., lower norepinephrine requirements or reduced vasoactive-inotropic scores) when integrated into individualized care pathways; however, effect estimates vary and certainty remains limited, arguing for cautious, targeted use and further randomized evaluation^4–6^. PS-DVB sorbents provide large internal surface area and predominantly hydrophobic interactions that favor largely non-specific binding of solutes in the middle-molecular-weight range (≈ 5–60 kDa). Importantly, this adsorption is selective with respect to molecular weight, hydrophobicity, and concentration, but not specific in the sense of ligand-directed capture (e.g., endotoxin adsorbers). This functional selectivity includes inflammatory mediators but also implies potential off-target adsorption of co-administered therapeutics^1–3^. Clinically, observational studies often report hemodynamic stabilization signals during hemoadsorption – such as reductions in norepinephrine dose or composite vasoactive-inotropic scores – yet randomized data remain sparse and overall outcome effects uncertain. A recent meta-analysis suggested possible short-term survival benefit but urged caution due to heterogeneity and study quality^4,7,8^. In parallel, a growing pharmacokinetic evidence base documents drug-specific removal of anti-infective agents by PS-DVB hemoadsorption, with in vitro and clinical work indicating clinically relevant adsorption for several antibiotics and antifungals; dose adjustment and therapeutic drug monitoring are therefore recommended in selected cases^9–13^. By contrast, catecholamines – the cornerstone of hemodynamic management in septic shock – remain poorly characterized with respect to direct removal by PS-DVB hemoadsorption. Prior research and reviews largely focus on antimicrobials and other drug classes, and we found no prior studies that directly quantified plasma epinephrine, norepinephrine, or dobutamine during PS-DVB hemoadsorption, despite frequent clinical reporting of reduced vasopressor requirements during treatment^4,11,12,14–20^. This gap motivated the present in vitro investigation to measure catecholamine concentrations systematically under controlled hemoadsorption conditions.

## Methods

**Fig. 1:**
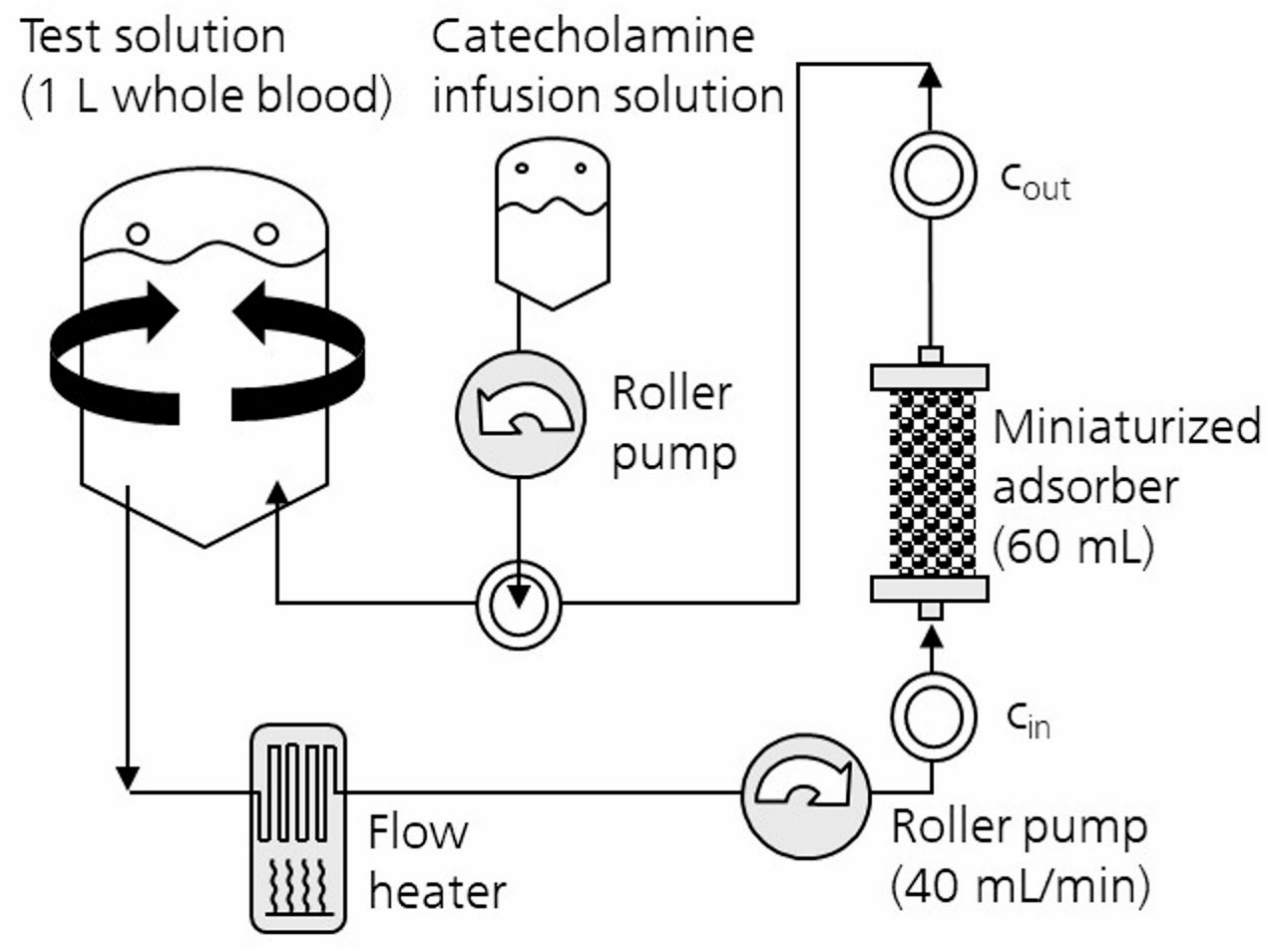
In vitro experimental setup. Samples were taken at the inlet (c_in_) and outlet (c_out_) of the miniaturized adsorber.

The experimental setup is shown in Fig. 1. The in vitro experiments were performed with miniaturized versions (60 mL sorbent volume) of the CytoSorb hemoadsorption column (CytoSorbents Europe GmbH, Germany). 1 L of EDTA-anticoagulated (K3EDTA) human whole blood mixed from two 500-mL blood bags of AB0- and rhesus-compatible healthy donors was used as test solution. This pool volume was chosen according to the ratio of 1:5 of the miniaturized device to the actual sorbent volume of 300 mL of the CytoSorb device assuming an average patient blood volume of 5 L to preserve the adsorber-to-blood-volume ratio of the clinical situation. The blood was donated max. 24 h prior to each experiment and stored at 4 to 5 °C until start of the experiments. To ensure undisturbed analyses of catecholamines, extended donor criteria were used, namely the donors were not allowed to consume alcohol, tea, coffee and nicotine12 h before blood collection and had to take a medication pause for 48 h before blood collection. A test blood pH value between 7.35 and 7.45 was adjusted by administration of Tris buffer solution (Dr. Franz Köhler Chemie GmbH, Germany). During the experiments, the pH was monitored using a blood gas analyzer and if necessary readjusted by further administration of Tris buffer solution every 30–60 min. A catecholamine bolus was added to the test blood at the start of each test run. Each catecholamine was tested in two independent experiments using fresh test blood (*n* = 2). Epinephrine, norepinephrine and dobutamine are known to be photo- and temperature-sensitive. Therefore, after an initial bolus, the substances had also to be administered continuously to the blood reservoir using a roller pump (REGLO ICC, Ismatec, Germany) to compensate for the concentration decrease by physicochemical decay over time. The decay rates (percentage concentration per minute) of each substance were determined in stationary batch experiments at 37 °C from a first-order kinetic fit of the respective concentration-time curve. The intended peak plasma concentrations (see Table 1) were oriented towards the therapeutic plasma levels of the respective substance in severe sepsis and septic shock. The initial substance bolus and the continuous infusion rate was calculated under consideration of the blood hematocrit, the recovery rate (determined in preliminary experiments, data not shown), and the substance decay rate. In the main experiments, the blood was circulated through the miniaturized adsorbers for 300 min with a blood flow rate of 40 mL/min using a laboratory roller pump (MCP Standard; Ismatec, Germany). The blood was warmed to 37 °C throughout the test duration with an infrared fluid and blood warmer (Fluido^®^; The 37 Company, Netherlands). The inlet and outlet pressure were monitored to detect critical pressure increases. All tubing and connectors for fluid transport were made from single-use medical-grade dialysis equipment (Heinz Meise GmbH, Germany). Samples were taken at the adsorber inlet and outlet after 0, 5, 15, 30, 60, 120, and 300 min.

**Table 1:** Target peak plasma concentrations of catecholamines in the test blood.

Catecholamine	Target in vitro plasma concentration	Brand name, dosage form, concentration
Epinephrine	20 ng/mL	Adrenalin 1:1000 Infectopharm, injection solution, 1 mg/mL
Norepinephrine	100 ng/mL	Arterenol 1mL, injection solution 1 mg/mL
Dobutamine	300 ng/mL	Dobutamin Liquid Fresenius, infusion solution, 5 mg/mL

The plasma clearance CL_Plasma_ was calculated for each time point as follows:

$$\:C{L}_{Plasma}={Q}_{B}*\left(1-Hct\right)*\frac{{c}_{in}{-\:c}_{out}}{{c}_{in}}$$

With the blood flow rate Q_B_ (= 40 mL/min), the hematocrit of the test blood (Hct) and the catecholamine concentration at the inlet (c_in_) and outlet of the miniaturized adsorber (c_out_).

To determine the decay of the drugs during the test period without continuous infusion, a small volume of the test blood was kept for 300 min in a falcon tube in a water bath at 37 °C under static conditions (control sample). The hematocrit in the test blood was determined with an automated hematology analyzer (XN-350; Sysmex, Japan). Samples for later substance analyses were transferred to tubes with glutathione to stabilize the catecholamines and centrifuged immediately. Plasma was collected carefully and stored at -20 °C until analysis.

Epinephrine and norepinephrine in plasma were analyzed at our laboratory with high-performance liquid chromatography (HPLC) and electrochemical detection using a commercial assay kit (RECIPE, Germany) with the internal standard 3,4-Dihydroxybenzylamine. Samples were prepared by means of a solid-phase extraction method (selective enrichment on alumina). For both catecholamines, the lower limit of detection and quantification was 8 ng/mL and 15 ng/mL, respectively, and the linearity range 15-2500 ng/mL.

Dobutamine in plasma was analyzed by an external laboratory (Medizinisches Labor Bremen GmbH, Germany) using a validated in-house method by HPLC and fluorescence detection (excitation 225 nm, emission 320 nm) after solid-phase extraction on C-18 phase (reversed phase). Concentrations and clearances are stated as mean +/- standard deviation.

## Results

**Fig. 2:**
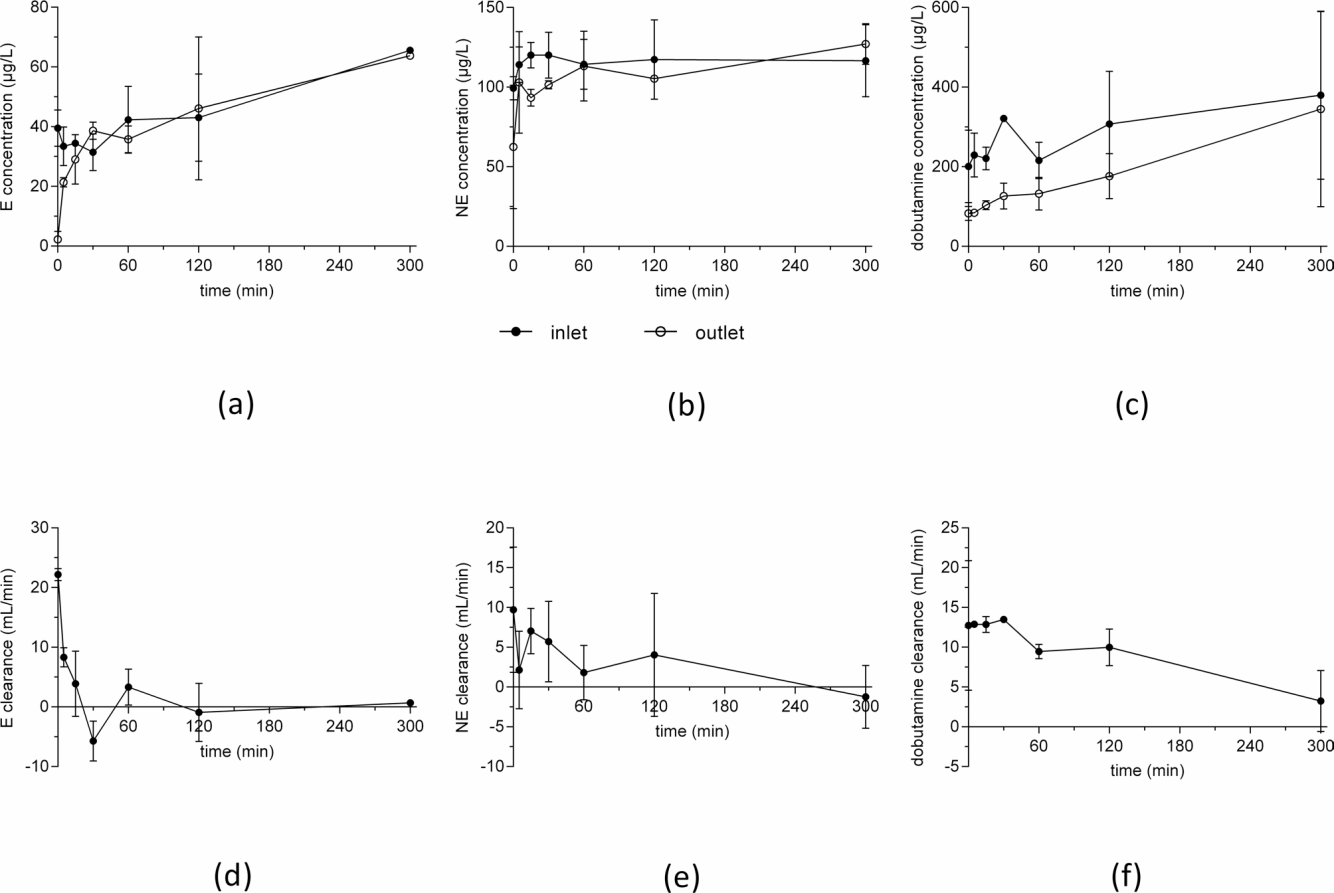
Concentration-time curves (**a**–**c**) and plasma clearances (**d**–**f**) epinephrine (E), norepinephrine (NE) and dobutamine during the in vitro experiments (mean ± standard deviation, *n* = 2).

In the present study, the impact of hemoadsorption with the CytoSorb column on plasma levels of different drugs was tested in an in vitro recirculation model. The in vitro decay rates of the catecholamines were determined to be 2.4%/min (dobutamine), 1%/min (norepinephrine) and 0.5%/min (epinephrine) from exponential fits of concentration-time curves observed in the preliminary tests. Almost constant concentrations and low clearance rates (< 5 mL/min) of epinephrine and norepinephrine indicate that only a minor removal of either substance occurs by the CytoSorb. In contrast, higher and consistent plasma clearances (5–15 mL/min) were observed for dobutamine (see Fig. 2).

## Discussion

Hemoadsorption with polystyrene-divinylbenzene (PS-DVB)-based cartridges has been increasingly adopted as an adjunctive extracorporeal therapy in patients with septic or vasoplegic shock, primarily to attenuate dysregulated inflammation and stabilize hemodynamics^5,21^. Against this clinical background, any relevant removal of vasoactive catecholamines would be undesirable. In the present in vitro study, epinephrine and norepinephrine concentrations remained essentially unchanged over 300 min, whereas dobutamine showed a reproducible apparent clearance of approximately 5–15 mL/min under otherwise identical conditions. These data indicate that, within the experimental system, PS-DVB hemoadsorption did not measurably remove epinephrine or norepinephrine but did produce detectable sorbent-mediated removal of dobutamine. Importantly, because this closed-loop in vitro model does not capture endogenous body clearance, the clinical relevance of the observed dobutamine removal cannot be inferred directly.

From a clinical perspective, the absence of measurable epinephrine or norepinephrine removal is reassuring and aligns with current observational evidence, in which hemoadsorption has not been associated with acute loss of vasopressor efficacy. Large single-center and multicenter cohorts of septic shock patients treated with PS-DVB consistently report reductions in catecholamine requirements and, in some analyses, improved survival, but without direct catecholamine pharmacokinetic measurements^4,22^. These hemodynamic improvements are generally attributed to mitigation of the inflammatory response and microcirculatory dysfunction rather than to direct manipulation of vasopressor levels^4,5,22,23^. Our in vitro findings are consistent with this interpretation: they suggest that reduced vasopressor doses during PS-DVB hemoadsorption are unlikely to be explained by direct removal of epinephrine or norepinephrine and instead more likely reflect a more favorable circulatory milieu. At the same time, the selective adsorption of dobutamine observed here indicates that inotropes may be more susceptible than classical vasopressors to sorbent interaction. Whether this translates into clinically relevant underexposure in vivo, however, remains to be determined.

The physicochemical interaction between catecholamines and PS-DVB provides a plausible mechanistic explanation for this differential behavior. PS-DVB sorbents are highly porous aromatic polymers with a large internal surface area and a polyvinylpyrrolidone coating; molecular uptake is driven predominantly by size and hydrophobic interactions within a target range of roughly 5–60 kDa, but smaller, sufficiently hydrophobic molecules can also be captured^21,24,25^. Experimental work with PS-DVB beads has demonstrated efficient removal of a broad panel of pathogen- and damage-associated molecular patterns, cytokines, mycotoxins and other amphiphilic mediators from whole blood, confirming that hydrophobicity and aromaticity strongly modulate adsorption beyond simple size cut-offs^9,24,25^. Our observation that dobutamine, but not epinephrine or norepinephrine, is adsorbed under otherwise identical conditions is therefore consistent with the notion that subtle differences in aromatic substitution and side-chain structure are sufficient to determine effective affinity for the PS-DVB surface.

Structurally, all three agents are catecholamines, but they differ in their side chains and resulting ionization and lipophilicity profiles. Norepinephrine and epinephrine are relatively small, highly polar molecules with primary amine groups that are fully protonated at physiological pH and show markedly negative distribution coefficients in aqueous buffer, indicating a strong preference for the aqueous phase^26–30^. Dobutamine, in contrast, introduces an additional arylalkyl moiety on the side chain, increasing aromatic surface and hydrophobic character despite remaining largely protonated at physiological pH. Quantitative chromatographic and computational studies of biogenic amines place dobutamine closer to the more lipophilic sympathomimetic drugs, whereas epinephrine and norepinephrine cluster among the most hydrophilic catecholamines^26,27,30^. It is therefore biologically plausible that dobutamine achieves sufficient local partitioning into the hydrophobic microenvironment of the PS-DVB pores to be measurably cleared, while epinephrine and norepinephrine, being more hydrophilic and strongly hydrated, show negligible affinity for the sorbent.

An important methodological question is whether the flat concentration-time profiles of epinephrine and norepinephrine under our experimental conditions truly reflect the absence of adsorption or could be masked by analytical or preanalytical artefacts. In our set-up, all catecholamines were administered as an initial bolus to rapidly achieve target concentrations, followed by a constant-rate infusion to approximate a pharmacologic steady state. Under such bolus-plus-infusion conditions, any additional extracorporeal clearance introduced by PS-DVB hemoadsorption would be expected to manifest as a lower plateau concentration and/or a progressive downward drift over time. Catecholamines are chemically labile, prone to oxidation and adsorption to surfaces; however, modern HPLC- or LC-MS-based assays, when combined with appropriate stabilization and handling protocols, provide robust and highly sensitive quantification in complex matrices^29,30^. Classical and contemporary stability studies have shown that plasma catecholamines are relatively stable for several hours at room temperature and during prolonged storage when standard anticoagulants and acidified conditions are used, with minimal spontaneous loss under controlled laboratory conditions^31–33^. Similar work in urine demonstrates that free catecholamines and their methylated derivatives remain stable across a range of storage times and temperatures when pH is adequately controlled^32,33^. In the present experiments, the close overlap and near-horizontal time courses of epinephrine and norepinephrine in the adsorber circuits, despite continuous infusion after the initial bolus, together with established stability data, make relevant assay drift, degradation, or hidden adsorption unlikely. Instead, they indicate that the PS-DVB sorbent has little or no effective capacity for these catecholamines at clinically relevant concentrations, even under conditions of sustained exposure.

By contrast, the differences of dobutamine concentrations at the inlet and outlet of the adsorber consistently observed throughout the experiments indicate an adsorber-related elimination pathway. As with epinephrine and norepinephrine, dobutamine was administered as an initial bolus followed by a constant-rate infusion; in a closed system without additional elimination, this design would be expected to yield similar inlet and outlet concentrations over time. Clinically, dobutamine is characterized by high systemic clearance and a short half-life^34^, but the present in vitro model does not allow the relative contribution of the adsorber-specific clearance to total body clearance to be quantified. This distinction is important. In the experimental in vivo study by Schneider et al.^9^, both total clearance and adsorber-specific clearance were calculated, and the relevance of CytoSorb-mediated drug removal was classified according to the percentage increase in total body clearance; only on that basis could removal be categorized as negligible, mild, or moderate. In contrast, our model can demonstrate mechanistic removal within the extracorporeal circuit, but it cannot determine whether the observed dobutamine clearance would represent a pharmacologically meaningful fraction of total clearance in patients. Accordingly, the present data support the conclusion that dobutamine interacts with the sorbent to a greater extent than epinephrine or norepinephrine, while the in vivo relevance of this interaction remains to be defined in dedicated clinical pharmacokinetic studies^9^.

These findings integrate well with the broader literature on drug–sorbent interactions during PS-DVB hemoadsorption. Systematic in vitro and clinical pharmacokinetic studies have shown that CytoSorb may exert little or no relevant effect on the exposure of many anti-infective agents, whereas a subset of drugs displays measurable additional adsorber-specific clearance^9,10,13^. In particular, Schneider et al.^9^ demonstrated in vivo that although most tested drugs showed some degree of adsorber-associated clearance, only a minority showed a meaningful increase in total body clearance once endogenous clearance was taken into account. In that study, the effect on total clearance was considered moderate for fluconazole and linezolid, mild for liposomal amphotericin B, posaconazole and teicoplanin, and negligible for all other drugs. This framework is highly relevant to the interpretation of our data. Our results add dobutamine to the group of substances for which PS-DVB hemoadsorption can produce measurable sorbent-mediated clearance, but, unlike the Schneider study, our in vitro design does not permit classification of that effect according to its proportional contribution to total body clearance. The present findings should therefore be interpreted as evidence of a mechanistic difference between dobutamine and the two endogenous catecholamines, rather than as proof of clinical relevance per se.

The therapeutic implications of these observations are therefore nuanced. For epinephrine and norepinephrine, the absence of measurable in vitro removal argues against any need for empiric dose escalation solely because a PS-DVB adsorber is present in the extracorporeal circuit. This is consistent with clinical data suggesting that vasopressor dose reductions during hemoadsorption are more likely to reflect improved vascular responsiveness and reduced inflammatory burden than device-mediated drug loss^4,5,22,23^. For dobutamine, however, our results support a more cautious and explicitly exploratory interpretation. Notably, dobutamine is routinely titrated to hemodynamic effect rather than to predefined target plasma concentrations, which may render modest extracorporeal removal—if clinically present in vivo—more readily correctable at the bedside than underexposure of exposure-driven drug classes such as anti-infectives or immunosuppressants. At the same time, the present data make reduced exposure due to extracorporeal adsorption biologically plausible. Prospective clinical pharmacokinetic studies should therefore directly quantify dobutamine concentrations during hemoadsorption and determine whether the observed sorbent interaction translates into a clinically meaningful increase in total clearance that would justify dose adaptation. Until such data are available, any decision to modify dobutamine dosing during hemoadsorption should remain guided by careful hemodynamic assessment rather than by a priori assumptions about drug loss.

Several strengths and limitations of this preclinical work must be acknowledged. A key strength is the use of a controlled in-vitro whole blood circuit with miniaturized PS-DVB adsorbers, which preserves the full cellular and protein milieu of blood and thereby captures sorbent-drug interactions under conditions that more closely approximate the clinical situation than buffer- or plasma-only systems. This complex setup inherently incorporates the influence of erythrocytes, leukocytes, platelets, plasma proteins and endogenous lipids, along with their associated effects on protein binding, micro-rheology and biocompatibility, and thus parallels prior mechanistic studies characterizing mediator removal and hemocompatibility of PS-DVB-based cartridges in whole blood^9,10,24,25^.

At the same time, important limitations remain. We studied only a single adsorbent type under defined flow conditions and with fixed catecholamine dosing; dynamic processes such as changing organ perfusion, metabolism, distribution into peripheral compartments, and bedside dose titration—known to shape catecholamine pharmacokinetics in vivo—are not reproduced in this closed-loop model^4,5^. The apparent clearance values reported here should therefore be interpreted primarily as evidence of the direction and relative magnitude of catecholamine–sorbent interactions within the circuit, rather than as direct quantitative surrogates of in vivo values. Although the sorbent and blood volume and the blood flow rate in the in vitro model were scaled to preserve the clinical sorbent-to-blood-volume ratio and contact time between blood and sorbent, miniaturization may alter adsorber geometry, flow behavior, and the relative contribution of tubing and circuit surfaces. A further limitation is that our model quantifies adsorber-related removal but cannot determine its contribution relative to total body clearance. This distinction is central to the assessment of pharmacological relevance: in the in vivo CytoSorb study by Schneider et al., adsorber-specific clearance was interpreted only after relating it to endogenous clearance and calculating the percentage increase in total clearance. Our study cannot perform that step for catecholamines. Accordingly, the transferability of our findings should be viewed asymmetrically: the absence of measurable removal in vitro likely argues against clinically meaningful removal in vivo, whereas detectable adsorption in vitro does not necessarily predict clinical relevance. Moreover, although we refer to PS-DVB hemoadsorption broadly, our experiments used a specific miniaturized CytoSorb device; potential differences in polymer architecture, pore-size distribution, and surface modifications across PS-DVB-based platforms limit generalizability, and removal of norepinephrine or epinephrine cannot be categorically excluded for all hemoadsorption systems. Finally, our conclusions are based on a limited number of experimental runs and should therefore be regarded as hypothesis-generating, particularly with respect to the quantitative magnitude of dobutamine clearance.

## Conclusions

In conclusion, this in vitro study shows that PS-DVB hemoadsorption did not measurably remove epinephrine or norepinephrine under the tested conditions, whereas dobutamine exhibited consistent sorbent-mediated removal. These findings provide mechanistic support for clinical observations of reduced vasopressor requirements during PS-DVB hemoadsorption that are unlikely to be explained by epinephrine or norepinephrine removal, and they highlight dobutamine as a potentially vulnerable inotrope in this setting. Taken together with the broader literature on drug-sorbent interactions, they underscore that extracorporeal hemoadsorption should be evaluated on a drug-by-drug basis, with particular attention to agents that are structurally more lipophilic or amphiphilic. Future clinical work should focus on defining dobutamine pharmacokinetics and pharmacodynamics during PS-DVB hemoadsorption and on developing pragmatic dosing strategies that preserve intended inotropic effects while maintaining the safety and potential hemodynamic benefits of adjunctive hemoadsorption therapy.

## Supplementary Information

Below is the link to the electronic supplementary material.

Supplementary Material 1

## Data Availability

All data generated or analyzed during this study are included in this published article and its supplementary information files.

## Abbreviations

PS-DVB: Polystyrene-divinylbenzene
Hct: Hematocrit
Q_B_: Blood flow rate
CL_Plasma_: Plasma clearance
HPLC: High-performance liquid chromatography
LC-MS: Liquid chromatography-mass spectrometry
c_in_: Catecholamine concentration at the inlet of the miniaturized adsorber
c_out_: Catecholamine concentration at the outlet of the miniaturized adsorber
